# Specification of BMP Signaling

**DOI:** 10.3390/cells8121579

**Published:** 2019-12-05

**Authors:** Joachim Nickel, Thomas D. Mueller

**Affiliations:** 1 Department of Tissue Engineering and Regenerative Medicine (TERM), University Hospital Wuerzburg, Roentgenring 11, D-97070 Wuerzburg, Germany; 2 Fraunhofer Institute for Silicate Research, Translational Center Regenerative Therapies (TLC-RT), Roentgenring 11, D-97070 Wuerzburg, Germany; 3 Department of Molecular Plant Physiology and Biophysics, Julius-von-Sachs Institute, University Wuerzburg, Julius-von-Sachs Platz 2, D-97082 Wuerzburg, Germany

**Keywords:** TGFβ/BMP signaling, ligand-receptor promiscuity, signal specification

## Abstract

Bone Morphogenetic Proteins (BMPs) together with the Growth and Differentiation Factors (GDFs) form the largest subgroup of the Transforming Growth Factor (TGF)β family and represent secreted growth factors, which play an essential role in many aspects of cell communication in higher organisms. As morphogens they exert crucial functions during embryonal development, but are also involved in tissue homeostasis and regeneration in the adult organism. Their involvement in maintenance and repair processes of various tissues and organs made these growth factors highly interesting targets for novel pharmaceutical applications in regenerative medicine. A hallmark of the TGFβ protein family is that all of the more than 30 growth factors identified to date signal by binding and hetero-oligomerization of a very limited set of transmembrane serine-threonine kinase receptors, which can be classified into two subgroups termed type I and type II. Only seven type I and five type II receptors exist for all 30plus TGFβ members suggesting a pronounced ligand-receptor promiscuity. Indeed, many TGFβ ligands can bind the same type I or type II receptor and a particular receptor of either subtype can usually interact with and bind various TGFβ ligands. The possible consequence of this ligand-receptor promiscuity is further aggravated by the finding that canonical TGFβ signaling of all family members seemingly results in the activation of just two distinct signaling pathways, that is either SMAD2/3 or SMAD1/5/8 activation. While this would implicate that different ligands can assemble seemingly identical receptor complexes that activate just either one of two distinct pathways, in vitro and in vivo analyses show that the different TGFβ members exert quite distinct biological functions with high specificity. This discrepancy indicates that our current view of TGFβ signaling initiation just by hetero-oligomerization of two receptor subtypes and transduction via two main pathways in an on-off switch manner is too simplified. Hence, the signals generated by the various TGFβ members are either quantitatively interpreted using the subtle differences in their receptor-binding properties leading to ligand-specific modulation of the downstream signaling cascade or additional components participating in the signaling activation complex allow diversification of the encoded signal in a ligand-dependent manner at all cellular levels. In this review we focus on signal specification of TGFβ members, particularly of BMPs and GDFs addressing the role of binding affinities, specificities, and kinetics of individual ligand-receptor interactions for the assembly of specific receptor complexes with potentially distinct signaling properties.

## 1. The SMAD Dilemma: Many Growth Factors but Just Two Principal Signaling Pathways

According to Miyazawa et al.: “TGF-β family ligands trigger signaling through hetero-oligomerization of two types of transmembrane receptors with intrinsic serine-threonine kinase activities: the type I and type II receptors. […] In the ligand-receptor complex, the constitutively active type II receptors phosphorylate and activate the type I receptors. The type I receptors then phosphorylate a subgroup of SMAD proteins, the receptor-regulated SMADs (R-SMADs). The R-SMADSs comprise SMAD2 and -3 for TGF-β and activin signaling, and SMAD1, -5, and -8 for BMP signaling. Phosphorylated R-SMADs form a heterotrimeric complex with a distinct common-partner SMAD (co-SMAD), SMAD4. The complexes then translocate to the nucleus, where they activate or repress gene expression in association with other transcription factors and transcriptional coactivators or corepressors (the SMAD signaling pathway)” [1].

**Figure 1:**
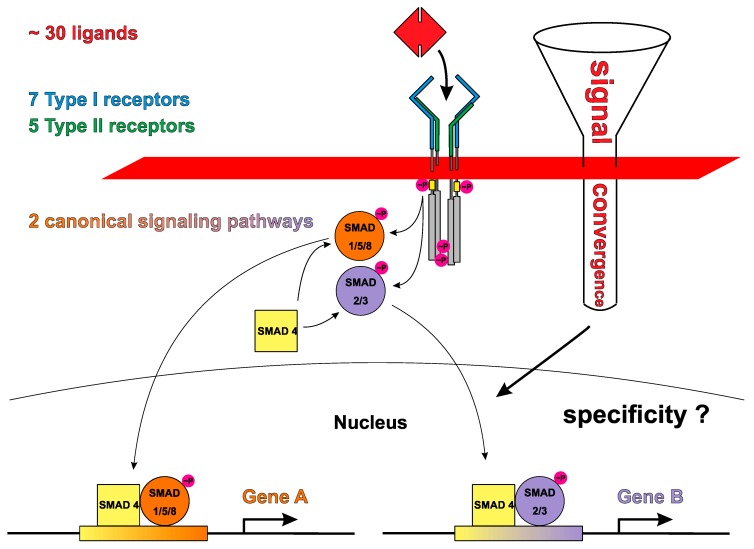
Usual depiction of the canonical TGFβ signaling pathways. This sketch neglects the presence of different receptors of either subtype as well as that of heteromeric ligands. Assuming that in this scenario the individual SMAD proteins of both branches, SMAD 1/5/8 or SMAD 2/3, are activated similarly, as a consequence a strong signaling convergence must be postulated. This results in a limited signal specification leading to the central question how these growth factors can then act as morphogens with highly distinct functions.

Numerous original papers and reviews during the past 20 years have introduced TGFβ/BMP receptor activation and signaling with these or very similar sentences (e.g., [2,3,4,5,6]). However, comparing the highly specific in vivo functions of the different TGFβ ligands as identified from animal studies with such a simple activation mechanism deduced from in vitro experiments or structure studies evidently raises the issue: How can this array of functions be derived from a receptor assembly mechanism that leads to subsequent activation of seemingly only two different (canonical) pathways, i.e., either the SMAD1/5/8- or the SMAD2/3 signaling cascade? (See also Figure 1).

It seems illogical that on the one hand Nature has diversified growth factors of this family to more than 30 known members, but at the same time restricted the signaling outcome of all ligands to initiate intracellular signaling pathways in just two different “flavors”. How Nature handles this obvious discrepancy and might produce ligand-specific signaling outcomes under these conditions, is still a focus of many research labs worldwide. One possible way out from this dilemma would be that what is slovenly described as SMAD1/5/8 (or SMAD2/3) signaling, does not constitute a single cascade in which all three (or the two) R-SMADs, i.e., SMAD1, SMAD5, and SMAD8, qualitatively and quantitatively deliver an identical signal, which would then invariably lead to identical gene transcription events for the three (or the two, respectively) SMAD factors. Initial characterization of the TGFβ/BMP pathway used classical in vitro tools to detect SMAD activation, i.e., antibodies detecting phosphorylation of conserved serine residues in the C-terminus [7,8] or reporter-gene assays using a minimal promoter element (e.g., BRE-luc [9] or CAGA-luc [10]). These are however incapable to discriminate between SMAD1, 5, and 8 (or between SMAD2 and 3) activation and can only specify the particular SMAD branch. SMAD proteins resemble classical transcription factors that contain a DNA-binding domain, i.e., the MH1 (Mad homology 1) domain, which is connected via a linker to a transactivation domain, i.e., the MH2 domain. SMAD1, 2, 3, 5, and 8, representing the R-SMADs, directly interact with type I receptors and are activated by those through phosphorylation at the C-terminus of their MH2 domain, i.e., the SSXS motif. They subsequently form heterotrimeric complexes with the shared SMAD4 via the MH2 domain and the phosphorylated SSXS motif. These complexes then act as transcription factors to regulate gene transcription. The specificity of the interaction between R-SMADs and type I receptors determines which R-SMAD branch is activated. R-SMADs 1, 5, and 8 associate with BMP signaling upon activation by the type I receptors activin receptor like kinase (ALK)1, ALK2, ALK3 and ALK6 and R-SMADs 2 and 3 are linked to activin and TGFβ signaling (as well as some GDFs) upon activation by the type I receptors ALK4, ALK5, and ALK7. This functional separation is backed by phylogenetic analyses clustering the R-SMADs into a SMAD1/5/8 and a SMAD2/3 branch [11]. Although SMAD proteins were found to be highly homologous (particularly within their MH1 and MH2 domains), the three and the two SMAD members within one branch do not share identical amino acid sequences thereby providing a possibility for a receptor-specific activation. Biochemical analyses, however, suggested that the specificity of the TGFβ/BMP type I receptor-SMAD interaction might be solely mediated by a short loop sequence in the receptor (L45 loop) and the R-SMAD protein (L3 loop), which differs only by a few amino acid residues between the type I receptors activating a different SMAD branch and two amino acid residues between SMAD1/5/8 and SMAD2/3 [7,12,13]. In addition, the L45 loop sequences show no amino acid difference between the type I receptors ALK3 and ALK6, which both activate SMAD1/5/8, or between ALK4, ALK5 and ALK7 known to activate SMAD2/3. This suggests that these type I receptors might not be able to differentially activate R-SMAD proteins of one branch [12]. Only the L45 loops of ALK1/ALK2 differ from that of ALK3/ALK6 indicating that ALK1 and ALK2 might activate R-SMADs of the SMAD1/5/8 branch differently compared to ALK3 and ALK6 [12]. Thus, ALK1/ALK2 might generate a different pattern of activated R-SMADs than ALK3/ALK6 which might provide the basis for further signal specification.

**Figure 2:**
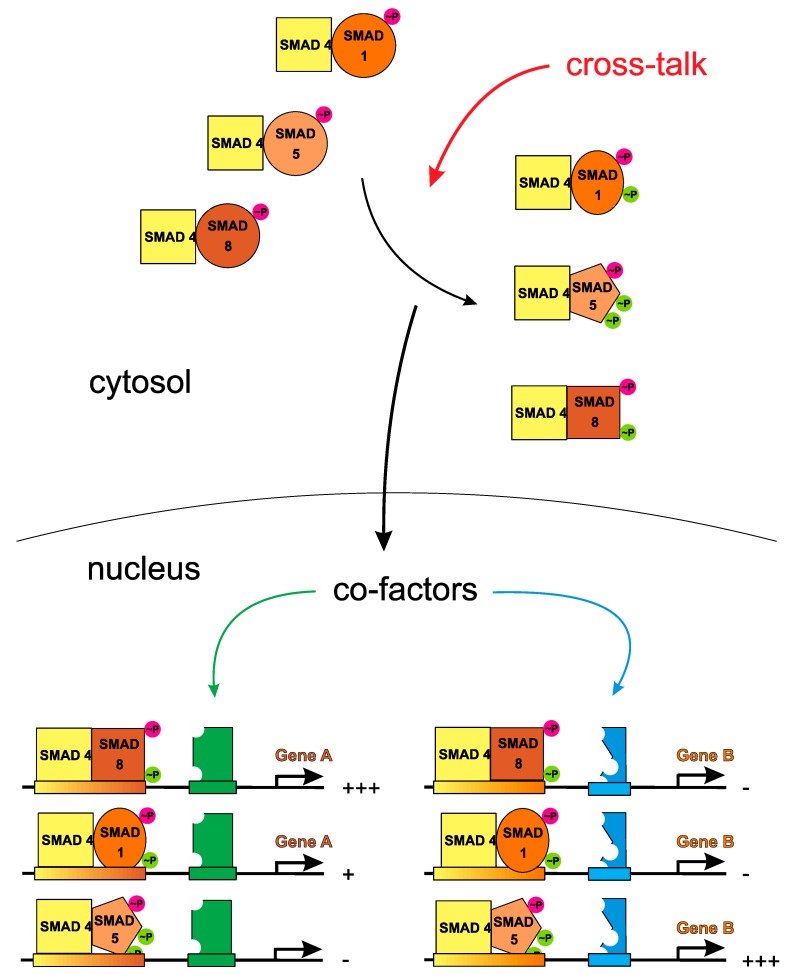
Specific interaction of particular SMAD proteins with transcriptional co-activators. Cytosolic interaction with other signaling cascades (cross talk) might produce R-SMAD/co-SMAD combinations interacting highly specific with distinct transcriptional co-activators. This allows the specific translation of signals induced by an individual TGFβ member thus resulting in a ligand specific regulation of a particular gene.

However, to make matters worse, structural analyses of complexes of SMAD MH1 domains bound to DNA, i.e., of SMAD1, SMAD2, SMAD3, and SMAD5 showed that the DNA-recognizing element, i.e., a β-hairpin harboring residues 75 to 82, is identical among all R-SMADs and engages in identical interactions with DNA [14,15,16]. While this remarkable finding might insinuate that all R-SMADs share similar DNA binding properties, one has to keep in mind that R-SMADs are acting as heterotrimeric complexes and differences in the architecture of these complexes can dramatically alter DNA recognition and binding. Unfortunately, no structure data are yet available for such larger full-length R-SMAD/Co-SMAD4 assemblies in complex with DNA making predictions on a mechanistic scale, how SMAD recognizes DNA to modulate gene transcription, impossible so far. The phosphorylation of R-SMADs in their C-terminal SSXS motif certainly describes the initial activation event in canonical TGFβ/BMP signaling, but multiple additional phosphorylation sites have been mapped in the DNA-binding MH1 domain and particularly in the linker region of R-SMADs (for review: [17]). While the sources for these phosphorylations are sometimes unclear (although involvement of different cytoplasmic kinases has been reported, e.g., cyclin kinases CDK8 and CDK9 [18]), phosphorylation of these additional sites seems to be ligand-dependent. Furthermore, other post-translational modifications, e.g., ubiquitylation, SUMOylation, acetylation, and ADP-ribosylation of R-SMADs have been observed, which can further diversify SMAD signaling (for review: [19,20]). Since the linker region in R-SMADs is highly variable (even within one SMAD branch), these modifications might reopen the possibility to encode a receptor-specific phospho-code (or modification code) to enable a TGFβ/BMP ligand-specific SMAD activation profile despite the limited number of R-SMADs (see Figure 2). That R-SMADs do indeed have specific functionalities/signals can be seen from animal studies employing conditional or systemic deletion of the various R-SMADs. Here distinct phenotypes were observed thereby indicating that R-SMADs of one branch do not necessarily (fully) compensate for each other, which would be a necessary consequence if all R-SMADs of one branch signal identically (e.g., [21,22,23,24,25,26,27]; for review: [28,29]).

Besides canonical SMAD signaling TGFβ/BMP ligands have also been reported to signal via a so-called SMAD-independent or non-canonical signaling pathways (for early reviews see. [30,31]). For instance, TGFβs were shown to activate different MAP kinase pathways, e.g., Erk, JNK and p38 [32,33,34,35], and similar observations were also made for BMP ligands [36,37,38]. Both, TGFβs and BMPs were shown to activate the TGFβ-activated kinase 1 (TAK1), which is a MAPKK kinase family member and is upstream of JNK and p38 [39,40,41]. Whether MAP kinase activation through TGFβs and BMPs is indeed fully SMAD-independent is a matter of debate as crosstalk between SMAD and MAP kinase signaling has been observed (e.g., [42,43,44]). However, most importantly, while the principal mechanism leading to canonical (also termed SMAD-dependent) TGFβ/BMP signaling is known, i.e., ligand binding leads to transphosphorylation in the type I-type II receptor complex leading to activation of R-SMADs via phosphorylation with subsequent formation of an R-SMAD/Co-SMAD assembly that translocates to the nucleus, almost nothing is known about the order of molecular events resulting in non-canonical TGFβ/BMP signaling. Furthermore, whether and how these are addressed in a ligand-specific manner is not yet understood, although it has been proposed that the nature of the ligand-binding receptor assembly may play a role [45].

## 2. The Ligand-Receptor Promiscuity Dilemma

While the additional post-translational modifications of R-SMADs described above might potentially establish a TGFβ/BMP-receptor specific R-SMAD activation code via a so far unknown mechanism, another observation in TGFβ/BMP receptor activation limits the possibilities for a supposed direct linkage between a particular TGFβ/BMP ligand and the encoded signal. In publications this additional dilemma is often stated as:

Weber et al. have stated that: “One important feature of the TGF-β superfamily is the limited specificity of its ligand-receptor interactions. For more than 30 ligands only seven type I receptors and five type II receptors are known. Thus, one receptor of a particular subtype has to bind several different ligands. But even though the ligands outnumber the available receptors, several BMPs and GDFs have been shown to interact with several different receptor chains of both type I and type II.” ([46]).

To yield a ligand-specific R-SMAD activation code each of the more than 30 TGFβ/BMP growth factors would have to address a specific combination of type I and type II receptor chains. Due to the limited number of receptors—only seven type I and five type II receptors serve the more than 30 ligands—most receptors usually interact with more than one TGFβ member though. In case of the type I receptors, which relay the ligand-receptor interaction into distinct R-SMAD:Co-SMAD complexes, this numeral discrepancy indicates that a given TGFβ/BMP member cannot yield a ligand-specific SMAD activation code if a receptor is utilized by more than one ligand (the limited number of receptors within this growth factor superfamily was recognized as early as 1992 [47]). To make matters worse, the above-described inevitable ligand-receptor promiscuity is aggravated by the fact that TGFβ/BMP members frequently bind to several TGFβ/BMP receptors of either subtype (for reviews: [48,49,50,51]). Hence, various TGFβ members likely form assemblies with identical receptor composition. This should inevitably yield identical intracellular signals, if these assemblies do not differ by other properties, e.g., architecture, or so far unknown additional components such as e.g., co-receptors. Ligand-receptor promiscuity was identified by interaction analysis using in vitro methods such as surface plasmon resonance and utilizing recombinant ligand and receptor proteins (for the latter the extracellular domains were used) (e.g., [52,53,54]). These measurements were often verified by cell-based assays, which analyzed the binding of radioactively labeled ligand proteins to ligand-responsive cell lines or to cells recombinantly expressing individual receptors [52,55,56]. As a result, out of the 12 type I and type II receptors serving the more than 30 TGFβ members only two seem to be ligand-specific or at least limited to a small TGFβ ligand subgroup. The Anti-Mullerian hormone type II receptor (AMHRII) is so far found to be highly specific for its ligand, Anti-Mullerian hormone (AMH, also known as Mullerian inhibitory substance MIS), and does not seem to interact with any other TGFβ member [57]. Due to its nature as a type II receptor its impact on SMAD signaling is unclear as SMAD activation is thought to be solely initiated from the type I receptors recruited into the ligand-receptor assembly. For AMH utilization of the TGFβ type I receptors ALK2, ALK3 and ALK6 was reported, all of which are shared with various BMPs, and thus AMH activates SMAD1, 5 and 8 seemingly similar as many other BMPs [58,59,60]. Thus, signal specification for AMH might be either due to the tissue-specific expression of its type II receptor, an AMHRII-specific activation (e.g., phosphorylation code) of the type I receptors involved or due to non-canonical, SMAD-independent signaling pathways that are initiated through AMHRII [57]. Besides AMHRII, only another type II receptor, the TGFβ type II receptor TβRII, shows a similarly high ligand-specificity. However, in contrast to AMHRII that binds a single TGFβ ligand member, TβRII interacts with all three TGFβ isoforms 1, 2, and 3 although binding strength considerably varies among the three isoforms [61,62]. Structure analyses of TGFβ ligand-receptor complexes then provided molecular clues as to why the TGFβ receptor TβRII only interacts with the three TGFβ isoforms. While TGFβ 1, 2, and 3 share the same ligand architecture with other TGFβ/BMP members the localization of the receptor binding sites differs (particularly for binding of the type II receptor TβRII) ([63,64], for review: [49]). In addition, binding of the type I receptor ALK5 to TGFβs was shown to depend on a direct interaction with TβRII [65]. These two observations might explain the specificity of the combination of these two TGFβ receptors for TGFβ1, 2, and 3, although earlier reports identified ALK5 as the potential type I receptor for the SMAD2/3-activating TGFβ member GDF9 [66]. While the exclusive usage of a TβRII/ALK5 combination by TGFβs indicates that at least the signal encoded by the sensu-stricto TGFβs might be distinct from other TGFβ members, studies have shown that TGFβ1, 2, 3 can activate the SMAD1/5/8 pathway in addition to its classical SMAD2/3 activation [67]. Although initial reports suggested alternative TGFβ-receptor assemblies comprising the SMAD1/5/8-activating type I receptor ALK1 [67,68], recent reports also suggest that TGFβs can initiate SMAD1/5/8 signaling by heteromeric assemblies comprising both type I receptors ALK2 and ALK5 [69].

**Figure 3:**
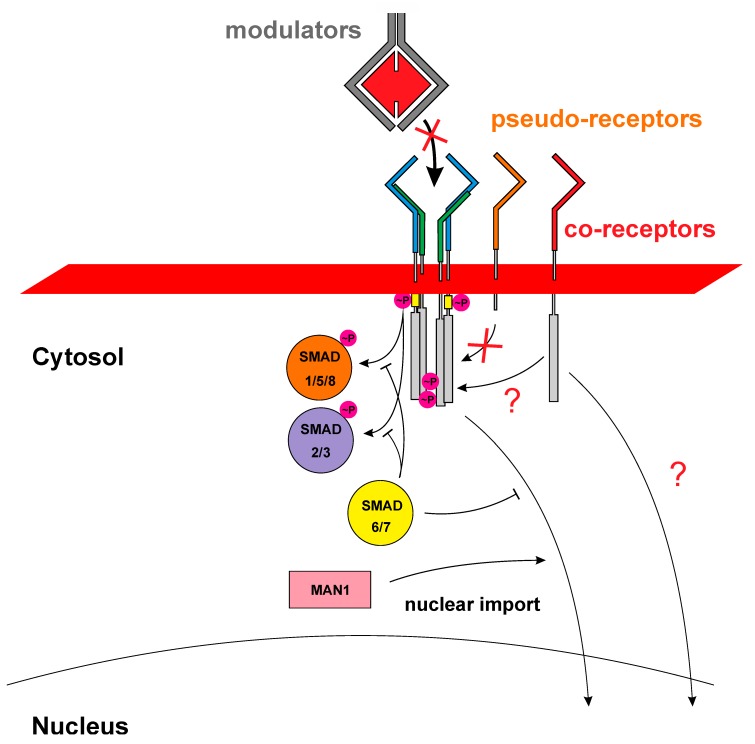
Mechanisms for specifying/modulating signal transduction of TGFβ family members. Signal transduction of TGFβ family members can extracellularly be regulated by interactions of the ligand with so-called modulator proteins. On the level of the cell membrane co- and pseudo-receptors exist either impeding, elevating or specifying signal transduction. In the cytosol signaling can be diminished/abolished by inhibitory SMADs (iSMADs) 6 and 7. Further signal specification can be added by controlling the nuclear import e.g., by Man 1 [73].

**Figure 4:**
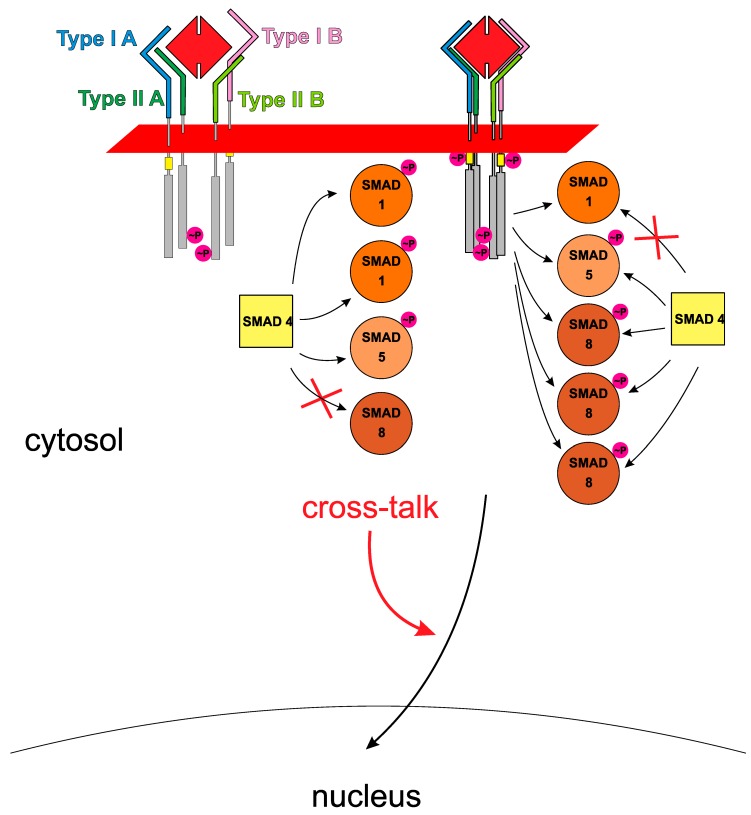
Alternative view on SMAD protein activation. This scenario involves the presence of up to four different receptor chains transmitting more individual signals after binding of homo- or heterodimeric ligands. If particular SMAD proteins of either branch are phosphorylated differently by individual type I receptors or type I/type II receptor combinations each ligand might generate specific combinations of activated SMAD proteins. The different SMAD proteins might now interact specifically with kinases/phosphatases of other signaling cascades.

Given the high ligand-receptor promiscuity it seems therefore imperative to establish: (a) signal specification-controlling mechanism(s) in order to provide for ligand-specific properties as found in vivo. In the past 20 years research of numerous groups have identified various different mechanisms that act either in the cytoplasm or extracellularly, here either located at the cell surface or the extracellular space (Figure 3). So-called modulator proteins such as noggin, chordin, follistatin or others interfere with binding of TGFβ ligands to their respective receptors (therefore often also termed antagonists) (for review: [49,70]). If these modulator proteins exhibit ligand specificities being different to those of the locally expressed receptors, which serve these TGFβ ligands, these modulator proteins can indeed specify signals when a mixture of TGFβ growth factors is present. However, as the modulator proteins are secreted proteins that do not have an intracellular domain capable to directly modulate the intracellular signaling cascade their effect on the transduced signal is rather indirect by (individually) altering the local active concentration of individual ligands. At the level of the cell surface, co- or pseudo-receptors can enable or alter the signaling capabilities of ligands in a subgroup-specific manner and if these co-receptors harbor a cytoplasmic domain a direct and ligand-dependent modulation of the transduced signal seems possible (for review: [71]). Also, in the cytoplasm further signal diversification can be achieved, for instance SMAD signaling can be inhibited or attenuated by inhibitory SMADs, i.e., SMAD6 and SMAD7. Additional proteins either interacting with the cytoplasmic domains of the TGFβ/BMP receptors or with R-SMAD proteins can modulate signaling by altering their phosphorylation status or adding other post-translational modifications (for review [20,72]). However, new mechanisms other than the current ligand-mediated receptor assembly may be necessary to explain how these intracellular modifications can discriminate between two different ligands forming the same assembly (see Figure 2 and Figure 4). As numerous reviews have focused on these types of signal diversification mechanisms we will not reiterate these aspects in this article. Instead, we would like to present intrinsic properties of the ligands and receptors of the TGFβ superfamily, e.g., binding affinities, binding kinetics, formation order and geometry of the ligand-receptor complex as possible source for signaling diversification. These parameters not only form the basis of the ligand-receptor interaction, but could also contribute to signal specification as these parameters influence the initial step of receptor activation and signal transduction.

## 3. The Beginning–Correlating Cellular Binding Sites and Receptors

Initial research investigating TGFβ signal transduction was performed using TGFβ ligands that were recombinantly produced in higher eukaryotic cells [74,75,76,77]. Protocols for purification of these recombinant TGFβ ligand proteins [78,79,80] as well as their radioactive labeling [52,81] were established, which was crucial to allow identification of cellular binding sites that could potentially contain the cognate cell surface receptors the scientific community was searching for. Following this concept and by using radioactively labeled TGFβ1 (purified from human platelets, [82]) or activin A (derived from recombinant expression in CHO cells, [83]), ligand binding sites could be identified on the surfaces of different cell lines. These sites were not only characterized in terms of binding, but this technique also provided affinities and could give additionally an estimate about the number of binding sites per cell. For TGFβ1 Massague and Like reported affinities ranging from about 50 to 90 pM and found about 20,000 binding sites per cell [84]. Similar numbers were published for activin A by Kondo et al. a few years later [83]. Here, by analyzing different cell lines about 3,500 to 6,500 binding sites per cell were reported for activin A with affinities ranging from 0.15 to 3 nM [83]. In both studies it was clearly shown that binding of TGFβ1 or activin A could not be competitively inhibited by any other ligand known at that time indicating that both TGFβ members interact with a highly specific receptor. Importantly, Scatchard analyses identified two different types of binding sites for activin A, which exhibited an approximately ten-fold difference in affinity for activin A and were thus termed “high” and “low” affinity binding sites. This observation highlights a general problem with this type of experiments as nothing can be said about the nature of the receptor as to whether it’s a single receptor molecule or a dimeric or even oligomeric receptor complex with defined architecture and oligomerization state. Furthermore, additional cell surface structures such as co-receptors or components of the extracellular matrix (e.g., proteoglycans) could participate in this interaction and thus the affinities and binding sites determined could represent an unknown structure, which might unfortunately be mistaken as the cognate receptor. In case of TGFβ1 chemical crosslinking indeed identified three proteins of different molecular weight contributing to the binding site identified on the cell surface. These were termed type I, II, and III receptors according to their size. Similarly, the two binding sites with different affinities for activin A also strongly indicated that either different “receptors” exist or that “receptor subunits” can form variable receptor assemblies.

Thus, to unravel the nature/architecture found on cells the receptors/receptor components had to be cloned and produced recombinantly in order to validate their function. The first TGFβ receptors that were cloned by time-consuming expression cloning approaches were activin type II receptor (ActRII) in 1991 by Mathews and Vale [85] and TGFβ type II receptor (TβRII) by Lin and co-workers one year later [61]. Both receptors were identified through screening of cDNA libraries. Subsequently, ligand binding assays were carried out with transfected cells overexpressing these receptors. Here binding specificities and affinities found for the interaction of activin A with ActRII recombinantly expressed on COS cells were identical to those obtained from radio-ligand binding experiments utilizing non-transfected, activin A-responsive cells. While these observations indicate that the cloned gene indeed encodes a/the receptor for activin A, several other explanations can be provided to also interpret these results. Firstly, these data might indicate that only one receptor for activin A exists, which is capable to transduce the signal into the cell on its own. In this case the stoichiometry of the activin A:ActRII interaction still remains unknown. Secondly, if a heteromeric receptor is responsible for activin A signal transduction, the second receptor must be expressed endogenously and no species specificity barrier must exist preventing the binding of the recombinant (in this case human) activin A protein to the endogenous receptor (subunit) in COS cells (*Chlorocebus aethiops*). However, using COS cells transfected with ActRII and performing chemical cross-linking experiments with radioactively labeled activin A yielded only a single band in gel electrophoresis while the same experiment performed with activin A-responsive cells showed an additional band with higher electrophoretic mobility. Considering the above-described data derived from the related TGFβ system, this observation rendered a potential mechanism in which ActRII acts as the sole receptor for activin A signaling implausible, despite the fact that cells transfected with ActRII showed the same affinity for activin A as non-transfected cells responsive for activin A. As a consequence, the second scenario that suggests a second receptor for activin A (a so-called type I receptor in analogy to the TGFβ system) best described all observations available at that time. However, as binding affinities were identical independent of the presence of the second receptor, either the second receptor does not interact directly with the ligand (which can be excluded from the observation of a cross-link product in gel electrophoresis) or its interaction with the ligand does not (significantly) contribute to the overall binding of the ligand to the receptor comprising all components.

In the following years modern techniques comprising new cloning strategies and the availability of luciferase-based reporter gene assays for direct readout of ligand-mediated receptor activation greatly facilitated the identification of further type I and type II receptors of this superfamily. The newly identified TGFβ receptors were usually named according to their biological function, e.g., the type I receptor for TGFβ signaling (TβRI). For type I receptors an alternative naming was established, which is based on their sequence homology to the original target, the activin type I receptor and hence denotes type I receptor as activin receptor-like kinases (ALK1 to ALK7) [86]. With these tools, ligand binding and ligand-mediated function of individual receptors or combinations of type I and type II receptor pairs could be examined. It is interesting to note that ligand-receptor binding often correlated well with ligand-mediated receptor activation as measured by reporter gene assays, but there have been reports in which binding and activity measurements were inconsistent. In such latter cases often binding of a ligand to a particular TGFβ receptor had been observed, while cell-based assays did not show receptor activation upon ligand exposure. Sometimes such observations unfortunately led to far-fetched misinterpretations of the data. For instance, in two early publications identification and functional characterization of a novel activin type II receptor (ActRIIB) and of two type I receptors were reported, which are nowadays known as ALK1 and ALK2 (also known as ActRI) [87,88]. Cells transfected with these type I receptors showed no cross-linking to radiolabeled TGFβ1 or activin A in the absence of the above-mentioned type II receptors. However, in the presence of activin type II receptors, ALK1 and ALK2 were cross-linked to radioactively labeled activin A, but not to TGFβ1. Reporter gene analyses (3TP-Lux; sensitive for SMAD2/3 signaling) revealed that the reporter luciferase was strongly expressed upon activin A stimulation in cells that were co-transfected with an activin type II receptors and either ALK1 or ALK2. With current knowledge most of these results must be considered incorrect, as both type I receptors are known to activate the SMAD1/5/8 pathway but not the SMAD2/3 branch, which however is the SMAD branch target of activin A. So, either the cell used for the reporter gene analysis endogenously expressed the correct activin type I receptor (ALK4) leading to the wrong assignment of ALK1 and ALK2 as activin A receptors or the SMAD reporter used here was too sensitive suggesting SMAD2/3 activation while in fact SMAD1/5/8 was activated.

Another example in which initial findings led to a premature conclusion was in the identification of receptors for growth and differentiation factor 5 (GDF5) [89]. Chemical cross-linking experiments identified the type I receptor ALK6 (also known as BMPRIB) as the exclusive type I receptor to interact with GDF5. The seemingly exclusive usage of ALK6 as demonstrated by these cell-based assays was then found to coincide with phenotypes in animal models in which either the *gdf5-* [90] or the *alk6/bmpr1b* [91] gene locus had been deleted. Based on this genotype/phenotype correlation, binding and functional properties of GDF5 were assumed to be strictly linked to this type I receptor. However, GDF5 can induce the expression of alkaline phosphatase (ALP) in the pre-chondrocyte cell line ATDC5 and does activate SMAD1/5/8 phosphorylation in the pre-osteoblastic cell line C2C12, although both cell lines do not express the type I receptor ALK6 [52,92,93,94,95,96]. This clearly indicates that GDF5 can transduce signals not only via ALK6, but similarly also through ALK3 albeit GDF5′s lower affinity for ALK3 might result in lower signaling efficiency. This is of importance as the tissue specific expression of ALK6 seems much more restrained than ALK3 and thus a strict coupling of GDF5 to ALK6 as the only signaling type I receptor would severely locally restrict GDF5 activity in vivo [89,97,98,99].

## 4. Do Type II Receptors Matter for TGFβ/BMP Signal Specification?

The two receptor subtypes exert mechanistically distinct functions during receptor activation: upon ligand binding at the extracellular side, the type II receptor kinase (which is considered constitutively active, although autophosphorylation of the type II receptor kinase seems to be required for full activity (see [17])) first phosphorylates the type I receptor kinase in a type I receptor-specific membrane-proximal glycine-serine rich domain termed GS-box. This then leads to activation of the type I receptor kinase, which subsequently phosphorylates R-SMAD proteins thereby initiating the canonical signaling cascade (see Figure 1). This sequential activation mechanism with a “non-constitutively active” type I receptor prior to activation by a type II receptor kinase was considered essential to enable a strictly ligand-dependent signaling mechanism (e.g., see [100]). In 1996 the Donahoe group showed that the immunophilin FKBP12 associates with TGFβ type I receptors and keeps them in an inactivated state [101]. Structural studies on ALK5 and later on ALK2 revealed the molecular mechanism of this interaction [102,103]. By binding to the GS-box, FKBP12 blocks the type II receptor kinase from accessing the phosphorylation target sites in the GS-domain and impedes a conformational opening of the bilobal kinase structure required for its activation. Consistently, mutations found in ALK2 of patients suffering from the heterotopic ossification disease FOP (Fibrodysplasia ossificans progressiva) are assumed to destabilize the inactive state leading to a (partially) activated ALK2 receptor kinase [102,104]. However, from the above outlined mechanism type II receptors only seem to have the task to activate the type I receptor kinase by phosphorylating a few key threonine and serine residues in the GS-box unique to type I receptors [105,106]. From this perception one could assume that any type II receptor could do this task as long as it indeed interacts with the given ligand. Thus, BMPRII as well as ActRII and ActRIIB, which interact with various BMPs/GDFs and activins, might be utilized promiscuously without affecting downstream signaling. That this assumption is too simple becomes readily evident from the fact that BMPRII contains a unique ~550 amino acid long cytoplasmic extension downstream of the intracellular kinase domain [107]. As an alternatively spliced short form, which ends after the kinase domain, similarly activates canonical SMAD signaling, a modulatory effect on type I receptor activation, which could alter SMAD signaling, seems unlikely [107,108]. Furthermore, numerous proteins, which were found to interact with the cytoplasmic tail of BMPRII, all seem to be involved in non-canonical signaling [109]. This might support the idea that BMPRII, ActRII, and ActRIIB activate a particular type I receptor in identical manner and hence do not influence canonical SMAD signaling. However, sequence analyses show a higher amino acid sequence variation in the kinase domains of the type II receptors compared to the type I receptors, which would argue for a greater variance in enzymatic properties, such as turnover number or substrate affinities and specificity in the type II receptor kinases. That not all type II receptors necessarily result in similar receptor activation despite binding the particular ligand was described in a study investigating GDF5 signaling [89]. In the original publication of Nishitoh et al. the strongest expression of the luciferase reporter gene upon stimulation with GDF5 occurred in cells that were co-transfected with ActRII and either ALK3 or ALK6 [89]. Lower but still significant luciferase expression was also detected in cells expressing BMPRII and either one of the above-listed type I receptors, although luciferase expression was rather weak for the combination BMPRII and ALK3. However more surprisingly, no GDF5-mediated reporter gene expression was found in cells in which either one of the type I receptors was co-transfected with ActRIIB, although chemical crosslinking experiments clearly confirmed binding of GDF5 to this type I-type II receptor combination [89]. The observation made by Nishitoh et al. presents a curiosity in that a receptor that binds to a TGFβ ligand with an affinity comparable to that for other receptors of the same subtype did not lead to signaling despite forming a similar ligand-receptor assembly as other GDF5 type I-type II receptor combinations. A similar observation was made by Perron and Dodd for BMP7 [110]. In their study of BMP7-evoked chemotaxis of monocytic cells they could show, that chemotaxis is mediated by the type II receptors ActRII and BMPRII, but not by ActRIIB [110]. It is important to note here that ActRIIB does not present a per se inactive type II receptor (that only functions as decoy) since it acts as activating type II receptor for the signaling of other TGFβ members such as activin A or GDF11 [111,112]. Since GDF11 and activin A activate SMAD2/3 and GDF5 and BMP7 signal via SMAD1/5/8 the effect of ActRIIB on TGFβ ligand signaling might be considered SMAD-branch dependent at first sight. However, Perron and Dodd showed that BMP7-evoked chemotaxis of monocytic cells is due to a non-canonical, SMAD-independent signaling and thus the different involvement of ActRIIB in TGFβ signaling follows a more complex mechanism [110]. A similar albeit indirect finding was also made by New and coworkers in a study investigating the different biological function of the activin type II receptors ActRII and ActRIIB [113]. Introducing mRNAs encoding for truncated ActRII or ActRIIB receptors (with the kinase domain deleted and thus acting dominant negative) into Xenopus embryos revealed that the truncated ActRIIB receptor caused axial defects. In contrast, the truncated ActRII receptors caused the formation of a secondary axes similar to the phenotype produced by inhibition of BMP4 signaling. Since this phenotype could not be established by the truncated ActRIIB receptor it indicates, that BMP4 does not transduce signals via this receptor. Our own experiments investigating type II receptor usage showed that also BMP2 did not activate SMAD1/5/8 signaling, if ActRIIB was co-transfected with ALK3 in COS cells, while ActRII and BMPRII in combination with ALK3 were capable to do so (unpublished data, Weber, D.; Sebald, W. and Nickel J.). This comes as a surprise as in vitro interaction analyses using surface plasmon resonance (SPR) showed that the extracellular domain of ActRIIB bound BMP2 (and also GDF5) with the highest apparent binding affinity compared to the other type II receptors although the differences between the three type II receptors were rather small (about ≤6-fold) [52]. But, what explanation can be provided that a ligand-receptor assembly consisting of BMP2, ALK3, and ActRIIB does not form an active signaling complex, while a complex in which ActRIIB is replaced by either BMPRII or ActRII, both of which share greater than 65% amino acid identity with ActRIIB, do so? Crystal structure analyses of two ternary complexes of BMP2 bound to ALK3 and ActRIIB (PDB entries 2H62 and 2H64, [46]) and to ALK3 and ActRII (PDB entry 2GOO, [114]) did not reveal any structural differences in the complex architectures that could explain different receptor activation. It should be noted that four alternative splice forms (termed B1 to B4) exist for the type II receptor ActRIIB [88]. These splice forms differ by inclusion of a short peptide segment (8 mer) in the extracellular domain just ahead of the transmembrane helix and/or another peptide insertion (24 mer) in the intracellular domain also located in close proximity to the transmembrane segment. Splice forms B1 and B2 both harbor the short segment in the extracellular domain, but differ in the presence or absence of the intracellular, juxtamembrane segment (B1 contains both insertions, while splice form B2 harbors only the extracellular insertion and thus closely resembles the type II receptor ActRII). The splice forms B3 and B4 both lack the insertion in the extracellular domain and similarly differ in the presence or absence of the intracellular splice segment. Radioligand binding of activin A to the four different ActRIIB splice forms revealed that splice forms B3 and B4 exhibited reduced ligand binding, while splice forms B1 and B2 that both contain the extracellular insertion segment did not show any difference in activin A binding compared to ActRII (for BMP4 differential binding to these different splice forms could not be observed, however it must be noted that the overall binding of radioactively labeled BMP4 to ActRIIB was rather low). This indicates that a removal of a short segment in the extracellular part close to the transmembrane segment significantly impairs activin ligand binding [88]. While the presence or absence of the intracellular splice segment did not affect activin A binding nothing is known regarding whether both splice forms differ in activin A-mediated receptor activation or downstream SMAD signaling. However, data from an animal model suggest that the ActRIIB B4 splice form, which lacks both splice insertions, can compensate for the other three splice variants and thus all four forms possibly present functional type II receptors [115]. In another study Liu et al. could show that in the osteoblast precursor cell line 2T3 BMP2 can induce SMAD signaling as well as expression of alkaline phosphatase via ActRIIB [116]. While the splice form of the ActRIIB receptor addressed in this study is not known, this observation might also point towards cell-type dependent functionality of ActRIIB. Although it is unclear from these limited data which role the type II receptor ActRIIB takes up in the signaling of different TGFβ members and by which mechanism these different effects are mediated, these examples break the simplification of all ligand-interacting type II receptor exerting the same function and which is often referred to in the following quote: “BMPs signal through two different types of serine/threonine kinase receptors. Three distinct type II receptors [BMP receptor II (BMPRII), activin receptor II (ActRII), and ActRIIB] and three type I receptors [BMPRIA, BMPRI1B, and activin receptor-like kinase 2 (ALK2)] have been identified. The mechanism of receptor activation involves BMP-induced phosphorylation of two sequentially acting kinases, with the type I receptor acting as a substrate for the type II receptor kinase. Activated BMP type I receptors relay the signal to the cytoplasm by phosphorylating their immediate downstream targets, SMAD1, SMAD5, and SMAD8 proteins.” [117].

Besides the fact that the potentially different functionality of ActRII and ActRIIB can possibly diversify the signaling outcome for a subset of BMP ligands, utilization of the activin type II receptors can add further complexity if different TGFβ/BMP ligands are present at the same time. Activin A and several SMAD2/3-activating GDFs, e.g., GDF1, GDF3, GDF8, GDF10, GDF11, also employ ActRII and ActRIIB to initiate downstream signaling. However, in contrast to most SMAD1/5/8-activating BMPs, such as BMP2, BMP4, BMP7, GDF5, etc., the SMAD2/3-activating activins and GDFs bind (in vitro) both activin type II receptors with considerably higher affinities (see e.g.,: [52,118,119]). Thus, the activin type II receptors can exert a dual signaling activity in a complex setting in which activin A and BMP2 (or a similar pair of SMAD2/3- and SMAD1/5/8-activating TGFβ ligands) are simultaneously present together with either activin type II- and their respective type I receptor. In the absence of BMPRII, activin A and BMP2 will directly compete for binding to the (shared) activin type II receptor. Since activin A binds ActRII with much higher affinity compared to BMP2, it will competitively impede the recruitment of activin type II receptors by BMP2. As a consequence, activin A will act as a competitive antagonist of BMP2 attenuating BMP signaling independent of the presence of the activin type I receptor ALK4 required for activin A signaling. A similar antagonism was also reported between inhibins and BMPs [120]. In the presence of the type I receptor ALK4, subsequent activin A signaling can provide additional signals (altered gene transcription profile), which dependent on the cell type can additionally antagonize canonical SMAD signaling induced by BMP2 [121,122]. Hence in such a setting, the active activin A signaling might attenuate BMP2 signals even more strongly than would be expected just from pure competition for binding to the shared activin type II receptor ActRII. Indeed, such antagonistic activities between activin A and BMP2 have been demonstrated in various biological contexts such as bone development and tumor biology [123,124].

## 5. BMP Type I Receptor Mysteries–Binding Does Not Mean Receptor Activation

Similar surprises regarding signaling complexity have also been observed for TGFβ/BMP type I receptors. Cell-based in vitro experiments measuring ligand binding and activation have identified ALK3 and ALK6 as activating type I receptors of BMP2 and BMP4 [125,126]. This nicely correlates with observations that both type I receptors bind BMP2 and BMP4 with the highest affinities among all type I receptors (e.g., [52]). Furthermore, BMP2 (and BMP4, data unpublished) binds the type II receptors ActRII, ActRIIB, and BMPRII with at least 50- to 60-fold lower affinity than the type I receptor ALK3. In addition, binding of BMP2 to all type II receptors is characterized by much faster kinetic rate constants (k_on_ and k_off_) compared to binding to type I receptors. Based on these pronounced differences it is assumed that BMP2-mediated receptor activation follows a sequential mechanism, in which the ligand BMP2 is recruited to the cell surface first by binding to its type I receptor. This intermediate binary complex then recruits type II receptors leading to receptor activation and subsequent initiation of downstream intracellular signaling. While GDF5 (as well as GDF6 and GDF7) does exhibit a similar receptor recruitment order as BMP2, type I receptor usage seems less promiscuous as for BMP2 (type II receptor interaction and specificity of GDF5 highly resemble that of BMP2 thus the main difference between BMP2 and GDF5 is the type I receptor specificity, see [52]). In vivo studies have indicated that the biological functions of GDF5 are coupled to ALK6 [90,91], although in vitro also binding of GDF5 to and SMAD signaling via ALK3 could be confirmed [89,93]. In order to explain this discrepancy, one might portend that GDF5 binds ALK3 with a 10-fold lower affinity than ALK6, which might not suffice signaling in vivo. However, in follow-up studies, mutations were identified that remove type I receptor specificity in GDF5 and GDF5 variants were developed that mimic BMP2 with respect to receptor binding [93,127]. Most importantly, such a variant, GDF5 R57A, despite binding ALK3 with identical affinity as BMP2 was still not capable to induce transcription of the same set of genes as BMP2 (please note type II receptor binding and specificity of GDF5 and this variant highly resemble that of BMP2) [127]. Whether this is due to a different SMAD activation profile by BMP2 and GDF5 R57A or a difference in non-canonical signaling of both growth factors is as yet unknown. However, this finding clearly indicates that different BMP/TGFβ ligands can assemble identical BMP/TGFβ type I-type II receptor complexes that do not necessarily deliver the same signal. That GDF5 indeed forms a ligand-receptor complex comprising ALK3 without subsequent receptor activation is confirmed by the observation that BMP2-mediated expression of alkaline phosphatase was attenuated by GDF5 (as well as GDF5 R57A) in a dose-dependent manner indicating a direct competition mechanism for the receptor [127]. The mechanistical difference that can lead to this differential activation by BMP2 and GDF5 is not yet known, but structure analyses did not reveal significant differences in the ligand-receptor assemblies [127]. Hence a simple mechanism that would involve structurally different complexes can be ruled out to explain the activation discrepancy. This is also in line with the observation that the difference between BMP2 and GDF5 in inducing alkaline phosphatase expression was cell-type specific. It would be very hard to imagine that BMP factors can establish BMP receptor assemblies with different 3D structures in different cell types.

Receptor activation by BMP6 and BMP7 showed another unexpected twist. Chemical crosslinking and cell assays identified ALK2 as the most efficient type I receptor for BMP6- and BMP7-mediated signal transduction [128,129]. Importantly however, both BMPs bind ALK2 in vitro with very low affinity (see e.g., [52,118,130]), while the two other SMAD1/5/8-activating type I receptors ALK3 and ALK6 interact with BMP6 and BMP7 with ≥30-fold greater affinities compared to ALK2 [52,130]. It thus seems odd that ALK2 would be efficiently recruited into a ligand-receptor assembly by BMP6/BMP7 when ALK3 and/or ALK6 are expressed at the cell surface at the same time unless their expression level is significantly lower. In a situation in which thermodynamic equilibrium would dictate the composition of the receptor assembly, one would assume that most complexes would harbor one of the two type I receptors with higher affinity. However, a structure-function study of BMP6 clearly showed that in the pre-chondrocyte cell line ATDC5 the lower affinity type I receptor ALK2 is required for induction of alkaline phosphatase expression. This confirms that ALK2 is recruited by BMP6 into a receptor complex for signaling despite ALK3 being also expressed in ATDC5 cells, which binds in vitro with 25-fold higher affinity to BMP6 [130]. Since ALK6 is not expressed in this cell line, no conclusion can be drawn regarding whether BMP6 can alternatively utilize ALK6 for signaling. Analyses of BMP6′ receptor binding properties showed that N-glycosylation at a site in the type I receptor epitope of BMP6 is essential for the binding of ALK2. This explains why bacterial-derived BMP6, which does not carry N-linked glycans, cannot bind ALK2. Since ALK3 and ALK6 do not require N-glycosylation for interaction, bacterially-derived BMP6 still binds to both type I receptors *in vitro*, but assembly of ALK3 containing complexes by BMP6 was found to not result in induction of alkaline phosphatase expression confirming the necessity of ALK2 for BMP6 signaling. However, when comparing the two closely related BMPs BMP2 and BMP6, it is not clear why BMP2 can assemble ALK3 into a signaling BMP type I-type II receptor complex while a similar interaction of ALK3 with bacterially-derived BMP6 does not initiate downstream signaling. While one might argue that BMP6 binds ALK3 much more weakly than BMP2, which might impede initiation of signaling via ALK3 this kind of argumentation seems preposterous given the fact that interaction of BMP6 with ALK2 is even weaker. Unpublished data from the Sebald lab suggests that signaling of BMP6 could be even more complex (see also [131]). Here, induction of ALP expression by glycosylated BMP6, non-glycosylated BMP6 and BMP2 were analyzed in the pre-osteoblast cell line C2C12 (these cells express the BMP type I receptors ALK2 and ALK3 but not ALK6; see [100,129]). In this experiment, ALP expression was induced by BMP2 and glycosylated BMP6, but not by non-glycosylated BMP6 confirming the hypothesis that BMP6 signaling requires recruitment of ALK2. Surprisingly however, ALP expression by glycosylated BMP6 could be down-regulated by an ALK3-neutralizing antibody (AbD1556 and AbD1564, see [132]) in a dose-dependent manner [131]. While for BMP2-mediated ALP expression this would be expected as BMP2 utilizes ALK3 as is known, the downregulation of BMP6-mediated ALP induction comes as a surprise as the above-described experiments already identified ALK2 and not ALK3 as signaling type I receptor of (glycosylated) BMP6. One explanation for this observation might be that (glycosylated) BMP6 assembles a heteromeric type I receptor complex in which ALK2 and ALK3 are both required for signaling. The ligand-dependent formation of ALK2-ALK3 heterodimers has been described recently to play a role in the regulation of hepcidin (a BMP6 target) in hepatocytes although the molecular mechanism of this type I receptor heterodimerization remains unclear [133].

Additionally, as consequence of the low affinity of BMP6 (as well as BMP7) for ALK2 it seems unlikely that these two BMPs are recruited to the cell surface via their interaction with ALK2. Instead BMP6 and BMP7 are possibly “anchored” to the cell membrane via the interaction with their type II receptors and these complexes subsequently recruit the type I receptor ALK2 to initiate signaling. Consequently, receptor assembly order of BMP6 (and BMP7) would be reversed compared to BMP2/BMP4 and could thus follow the same sequence as observed for activin A and most SMAD2/3-activating TGFβ ligands. Although it is not clear whether this will alter SMAD signaling of BMP6/BMP7 in comparison to that of BMP2/4 theoretical considerations suggest that reversal of receptor recruitment order could potentially influence downstream signaling at least in a quantitative manner. In the receptor recruitment scheme of BMP2 dissociation from the type I receptor is so slow that each particular ligand will likely activate only two type I receptors (i.e., due to the dimeric nature of the BMP ligand) and thus one ligand molecule will basically yield one activation signal. For BMP6/BMP7 (as well as TGFβ ligands which bind type I receptors with low affinity) the activated “low-affinity” type I receptor might dissociate from the membrane-located BMP-type II receptor complex to be replaced by another type I receptor, which might then get activated too. Hence, TGFβ ligands with this type of receptor recruitment order could activate multiple type I receptors per ligand-type II receptor assembly and thus a signal amplification might be possible for such ligands. Such an amplification mechanism would nicely explain the extreme sensitivity of some cell lines to TGFβ ligand exposure with half-maximal effective concentrations (EC50) far (in orders of magnitude) below their receptor affinities (K*_D_* values). For instance, growth of mink lung cells is blocked by the addition of TGFβ2 with EC50 values in the low pM range (0.6 pM ~ 2 pM) [134], whereas SPR measurements revealed binding affinities for the TGFβ2:TβRII interaction with K*_D_* values in the low nM range (5nM) [135].

This different receptor recruitment order might not only exert quantitative effects but could also lead to qualitative differences in signaling. As the receptors comprise a kinase activity, different lifetimes of the receptor in the assembly could result in different phosphorylation patterns in the cytoplasmic domains of the activated receptors dependent on the enzymatic properties, i.e., Michaelis-Menten constant (“affinity”) and turnover number (conversion rate). A type I receptor that dissociates fast from the BMP ligand-receptor assembly might not be phosphorylated at all sites compared to a type I receptor whose dissociation rate is slow. Differences in the receptor recruitment order and ligand-specific residence times of the individual type I and type II receptors in the ligand-assembled complex might thus enable a fine-tuning of receptor activation leading to differences in the encoded signals.

## 6. Heterodimeric Ligands–An Unexplored World

The above-described observations on BMP6 signaling already provided a hint that BMPs might be principally capable to assemble heteromeric receptor complexes harboring different receptors of each subtype thereby leading to receptor assemblies that contain two different type I receptor and/or two different type II receptors. While the homodimeric nature of TGFβ/BMP ligands rather seems to argue for symmetric receptor assemblies, similar binding affinities of TGFβ/BMP ligands for different receptors of one subtype, also known as ligand-receptor promiscuity, can however allow for assemblies consisting of four different receptors of which two belong to subtype I and two belong to subtype II (see Figure 4). Formation of such asymmetric receptor assemblies might be enforced by heterodimeric TGFβ/BMP ligands, which contain two distinct epitopes for type I receptor and two distinct sites for type II receptor interaction. However, experimental evidence for naturally occurring heterodimeric TGFβ ligands unfortunately is sparse except for the members of the Activin/Inhibin subgroup ([136], for review [137]). Direct experimental proof on the existence of naturally occurring heteromeric BMP ligands has so far been published for fish and fly [138,139,140,141]. Studies employing heterodimeric mammalian BMP ligands were therefore performed with recombinantly produced proteins. Due to the disulfide-linked dimer architecture that impedes formation of heterodimers outside the producing cell, the heterodimeric BMPs usually had to be produced by simultaneous co-expression of two BMP ligand genes (e.g., [142,143]). There is one exception to this rule, i.e., BMP15 and GDF9, which both lack the cysteine residue involved in intermolecular disulfide formation. Hence BMP15 and GDF9 can form heterodimers from homodimeric BMP15 and GDF9 in the extracellular lumen even when secreted from different cells [144]. The resulting BMP15:GDF9 heterodimer was found to exert unique biological functionalities (termed synergistic functions) not present in the homodimeric growth factors and that might be related to the fact that the heterodimer can simultaneously activate the SMAD2/3 (through its GDF9 subpart) and the SMAD1/5/8 (through its BMP15 subpart) branch [144,145,146,147].

A number of studies have investigated also other heterodimeric BMPs, mostly BMP2/6, BMP2/7, and BMP4/7, which were recombinantly produced and purified from co-expression in eukaryotic cell culture or from expression in bacteria and subsequent refolding [142,143,148]. A common observation of these studies was the strongly increased activity of the heterodimeric BMP proteins (i.e., lower half-maximal effective concentrations required to observe similar transcription levels of marker genes) compared to their homodimeric paralogues [143,148,149,150,151,152,153]. Different mechanisms were proposed to explain how these increased bioactivities might be exerted. One possibility might be the assembly of asymmetric receptor complexes that harbor different type I and type II receptors as suggested above (see Figure 4) [154]. For the type II receptor interactions such possible heteromeric assembly could be directly inferred from the type II receptor specificity of the related homodimers as the complete type II receptor epitope is formed within one ligand monomer [50]. The situation is however different for the type I receptors as both ligand monomers contribute to the formation of one type I receptor binding epitope and thus a novel type I receptor epitope will be created in the heterodimer not identical to either one of the related homodimeric BMPs [50]. Hence it is not clear how type I receptor specificity/specificities and affinities will be affected in such BMP heterodimers. Unfortunately, there are yet no studies published that investigated receptor binding parameters in heterodimeric BMPs in a quantitative manner. Unpublished data from the Sebald lab however indicated that the heterodimeric BMP2/6 and BMP2/7 bound ALK3 in a very similar manner as homodimeric BMP2, i.e., with high-affinity in the low nanomolar range (see also [131]). Most importantly, the bacterially-derived (hence non-glycosylated) heterodimeric BMP2/6 did not seem to bind ALK2 and this finding was thus consistent with the hypothesis that ALK2 binding requires N-glycosylation in BMP6, which cannot be present in bacterially-derived BMP2/6. Despite the inability of bacterially-derived BMP2/6 to bind ALK2, the heterodimeric BMP could nevertheless very efficiently induce expression of alkaline phosphatase (ALP) in cell types that could not be stimulated with bacterially-derived homodimeric BMP6. This suggests that the enhanced activity of bacterially-derived BMP2/6 is not necessarily a consequence of simultaneous binding of two different type I receptors as suggested above, but due to other so far unknown mechanisms. For instance, Little and Mullins proposed that the enhanced bioactivity of the BMP2/6 heterodimer is due to the simultaneous presence of a high-affinity binding site for a type I receptor, here ALK3 (derived from the “BMP2 site”), and a high-affinity binding site for a type II receptor, i.e., ActRIIB (derived from the BMP6 monomer subunit) [154] (which could be confirmed by in vitro binding analyses [155]). Consistent with this hypothesis, Seeherman et al. presented a strategy to create “hyperactive” BMPs with maximal bone restoration capacity [156]. Here, instead of utilizing a BMP heterodimer, the authors designed different activin/BMP chimeras with tailored type I and type II receptor binding properties. These homodimeric chimeras that comprised elements of BMP2, BMP6 and activin A showed high affinity binding to all three BMP type I receptors (ALK2, ALK3 and ALK6) as well as to all three type II receptors, i.e., BMPRII, ActRII and ActRIIB [156]. As anticipated these chimeras exhibited significantly greater bioactivity than the wildtype BMP analogs in vitro and in vivo and performed on par or even better than the BMP2/6 heterodimer. Although this observation might indicate that the elevated activities are due to high-affinity binding of both receptor subtypes we cannot rule out that this capacity is achieved through the assembly of different receptors of either subtype since these “artificial” chimeric growth factors were highly promiscuous and could bind various receptors of either subtype with seemingly identical affinity. It is important to note that the above-described example of heterodimeric BMP15:GDF9 clearly suggests that asymmetric assembly of different type I and different type II receptors not only has quantitative effects, e.g., higher activity than observed for the homodimeric analogs, but can also alter the gene transcription profile (possible mechanism is depicted in Figure 2 and Figure 4). Hence such asymmetric receptor complexes might encode unique and distinct functions not observed with symmetric receptor assemblies and thereby provide for signal diversification on basis of combinatorial receptor usage. Unfortunately, detailed gene expression analyses to compare the transcriptional profile of heterodimeric ligands with those from their homodimeric relatives have not yet been done. Importantly, the above-described example of BMP6 signaling suggests that asymmetric receptor assembly formation is not necessarily limited to heterodimeric ligands but could also be initiated by homodimeric ligands. Thus, to determine the “contribution” of each receptor to ligand signaling gene expression analysis should be performed using a panel of neutralizing antibodies raised against each of the TGFβ/BMP receptors to individually cancel participation of each receptor in the ligand-receptor assembly.

Finally, one might ask whether in mammals heterodimeric TGFβ/BMP ligands have a real physiological significance at all as the above-listed examples exclusively report from recombinantly produced BMPs. However, existence and occurrence of heterodimeric TGFβ/BMP ligands might be highly underrated due to lack of published data which again might be related to difficulties to experimentally detect these heterodimeric forms (particularly in the presence of homodimeric BMPs). Two older publications from the groups of Sampath and Wozney provided experimental proof for the existence of heterodimeric BMPs in mammals, however, not much further evidence has been added since then [157,158]. Recently new reports were published confirming the presence and function of heterodimeric BMP ligands in mammals [159,160]. These articles for the first time also describe novel and unique functions for such heterodimeric BMPs that cannot be exerted by a single homodimeric analog or a combination of both wildtype BMPs indicating that formation of heteromeric ligands can increase the signaling function and diversity of this protein family. This raises the question about the frequency with which heterodimeric TGFβ/BMP ligands occur and in which possible combinations they naturally exist. Considering that simple co-expression of two BMP genes was found to be sufficient for recombinant production it is unclear whether restrictions exist that would allow only heterodimer biosynthesis of certain combinations of TGFβs/BMPs. One potential mechanism that could facilitate or impair biosynthesis of certain heterodimers is in the biosynthesis of TGFβ/BMP ligands as precursor proteins consisting of a large N-terminal prodomain and a smaller C-terminal mature domain that harbors the activity of the TGFβ/BMP ligand. Upon secretion the prodomain is cleaved by proteolysis by metalloproteases of the furin family but in certain TGFβ/BMP ligands remains stably associated in a non-covalent complex with the mature domain to downregulate the bioactivity of the latter in a process termed latency (for review see [49]). Release of the mature C-terminal growth factor domain in these cases is then due to an active process regulated for instance by interaction with integrins. Structure analyses of such TGFβ/BMP proprotein complexes showed that parts of the prodomain of one monomer subunit fold into and interact with parts of the other monomer ([161,162,163], in particular see: [164]). Hence in heterodimeric TGFβ/BMP proprotein complexes such interaction can either stabilize and thus facilitate heterodimer formation or by destabilizing impair heterodimer folding and formation.

While it is thus unclear how many different heterodimeric BMP ligands exist and to what extent, it must be assumed that endogenous heterodimeric TGFβ/BMP ligands play a role in mammals. This has important consequences as many functions of TGFβs and BMPs on an organismic level were derived from knockout animal models (see review: [165]) and the phenotypes observed were solely attributed to a loss of the respective homodimeric ligand protein. However, assuming that knocking out a single TGFβ/BMP gene will also result in the loss of (several) heterodimeric TGFβ/BMP ligands certain functions might have been misaddressed to the homodimeric TGFβ/BMP analogues.

## 7. Conclusions

Observations and data summarized in this review indicate that our understanding of the molecular mechanisms of TGFβ/BMP signaling is still rather limited. From introductory descriptions in various original publications and reviews one might suppose a rather simple activation mechanism for TGFβ members in which the ligand (usually presumed homodimeric) assembles a symmetrical receptor complex comprising two type I and two type II TGFβ/BMP receptors of the same kind (as hypothesized from structure studies) leading to the activation of one of two principal downstream intracellular signaling cascades, i.e., SMAD1/5/8 or SMAD2/3. In these two principal cascades SMAD1, SMAD5 and SMAD8 (or SMAD2 and SMAD3 for the other group of ligands) are used synonymously and therefore termed SMAD1/5/8 (or SMAD2/3) potentially implicating that all R-SMADs of each branch deliver equivalent signals on transcriptional activation level. While this simplistic view somehow suggests a strongly converging signaling in which the more than 30 ligands through utilizing a too small set of only 12 receptors feed into just two signaling cascades, the diverse functions found for the more than 30 TGFβ members in vivo form a clear contrast to this impression (see Figure 1). The pronounced ligand-receptor promiscuity observed for numerous TGFβ/BMP ligands in vitro would further aggravate this signaling convergence problem as multiple ligands of the TGFβ family can form receptor assemblies with identical receptor composition, which should consequently deliver the same signals and hence encode identical functions, opposite to what is observed. Here we showed that while a ligand can bind several different receptors of either subtype (even if this occurs with comparable affinities) not all of these combinations necessarily deliver the expected receptor activation and signal. Such puzzling observations were made for type I as well as for type II receptors. Combinations of TGFβ type I and type II receptors that yielded a signal with a particular TGFβ member were found silent if assembled by a different ligand of the same TGFβ subgroup. That indeed the same receptors were assembled in these experiments could be reasoned from the fact that ligands could antagonize each other by competing for receptor binding. Thus (promiscuous) ligand-receptor interaction determined in vitro should not be mixed with (uniform) receptor activation. Unfortunately, we cannot provide a proven mechanism explaining for this surprising finding. 

One possible mechanism could be different assembly lifetimes that are due to different receptor affinities of the different ligands. As the receptors function as enzymes (kinases with possibly distinct enzymatic parameters, i.e., K_M_ and k_cat_) different receptor complex lifetimes may translate into distinct phosphorylation patterns either in the receptors themselves and/or in the intracellular (protein) substrates (one of which are the R-SMADs) thereby leading to different activation states. Similarly, receptor recruitment order, i.e., which receptor subtype is bound first and remains in complex with the TGFβ ligand at the cell surface until endocytosis, could influence the activation status/degree of the receptor as well as that of downstream targets. Thus, a more intelligible concept would be not to consider TGFβ receptor activation to work like a two-state on/off switch (which is always identically activated once the complex is assembled), but to look at the slightly different binding properties of the various ligands as a biologically significant intrinsic property that can be translated into distinct activation profiles. However, studying such details, e.g., ligand binding affinities or enzymatic properties of the receptor kinases, has been and still is regarded as nit-picking and thus systematic investigations have not yet been performed to figure if and how such differences modulate signaling.

In addition, the chemical nature of TGFβ ligands in vivo is unclear. As dimeric proteins, TGFβ ligands were and still are considered to exist as homodimers (mostly) although recombinant production highlights the simplicity with which heterodimeric TGFβ/BMP growth factors can be obtained from expression in eukaryotic cells. It is thus not known which and to what extent heterodimeric TGFβ/BMP ligands are endogenously produced in the different organisms, but it seems at least reasonable to assume that such heteromeric growth factor species occur naturally in many species. In the past many of the in vivo functions of TGFβ members that were deduced from animal models (transgenic of knockout) have been associated solely with the homodimeric forms, neglecting the possibility that some of these functions might originate from heterodimeric ligand species, which were “co-addressed” by the genetic manipulation. Hence, functionalities that cannot be reproduced by recombinant TGFβ/BMP proteins in vitro might be due to false assignment and could be a result from a heterodimeric species instead. While studies using recombinant heterodimeric TGFβ/BMP ligands have revealed strongly enhanced signaling activities and unique functions the molecular mechanism by which their signaling differs from that of related homodimeric ligands members is unclear. From the inherent asymmetry of heterodimeric TGFβ ligands enhanced formation of heterotetrameric receptor assemblies that harbor two different type I and/or two different type II receptors has been proposed as molecular cause for enhanced activity and altered signaling. However, whether this is indeed due to different kinase domains that might exhibit different substrate specificities or due to enhanced binding/stability of the assembled receptor complex is not known. While asymmetric receptor complex formation seems certainly more intelligible for heterodimeric TGFβ ligands, the above example of BMP6 signaling shows that assembling heterotetrameric receptor complexes is not limited to heterodimeric ligands.

Finally, statements that SMAD signaling has two branches, i.e., SMAD 1/5/8 and SMAD 2/3 might be misconstrued such that all TGFβ members utilizing SMAD 1/5/8 can uniformly activate any of the three R-SMADs with identical outcome for gene expression (the same would be assumed for SMAD 2/3-activating TGFβ members). However, tools used to analyze SMAD activation, e.g., antibodies binding to the phosphorylated C-terminus of the SMAD proteins, can only discriminate between the two branches, i.e., SMAD 1/5/8 or SMAD 2/3, but cannot specify the particular nature of the activated SMAD (or whether the different SMADs of one branch are differently activated) due to the high sequence similarity within the phosphorylation motif detected by the antibody. Similarly, analysis of SMAD signaling through measuring reporter gene expression is done by using an artificial promoter harboring one or several SMAD-binding elements that cannot discriminate between SMAD 1, 5 and 8 (or between SMAD 2 and 3). Hence, no specification can be deduced as to whether and which R-SMAD might be preferentially utilized by a particular ligand-receptor assembly on a cell. Similarly, nothing is known about the gene expression profile of a particular R-SMAD factor. R-SMAD proteins are multidomain proteins that heterotrimerize together with a Co-SMAD thereby forming the core of transcriptional regulation. Besides the two highly conserved MH1 and MH2 domains that engage in similar SMAD-SMAD or SMAD-DNA interactions, all five R-SMADs have a very distinct linker domain between the MH1 and MH2 domain that is subject to strong post-translational modification, e.g., phosphorylation by other kinases. In addition, SMAD proteins also interact with numerous other transcriptional co-activators and repressors. Thus transcription-mediating SMAD complexes can be highly diverse depending on the activating receptors and depending on the cellular context. This could lead to ligand-/context-specific gene expression profile explaining the highly diverse TGFβ/BMP ligand functions observed in vivo.

In summary, the above-listed observations suggest that our astonishment about the conflict between the highly diverse in vivo functionalities of the TGFβ ligands and a simplistic receptor mechanism utilizing a far too small set of receptors funneling into just two distinct pathways might be due to a mis-/overinterpretation of the available data. Considering the above examples, we have to admit that our current knowledge still lacks too many details about the molecular mechanism of TGFβ/BMP receptor activation and downstream signaling. While demanding additional novel components to participate in the ligand-receptor assembly, e.g., co-receptors, could be one route to ensure signal specification, yet undervalued differences in the intrinsic properties of the various known components, i.e., differences in the composition of the ligand-receptor assemblies, ligand-receptor affinities, etc. could also provide with distinct activation states that might be translated into ligand/receptor-specific gene transcription profiles. Understanding these mechanisms is crucial if we want to design TGFβ/BMP ligands with tailored functionalities. Such “2^nd^ generation” TGFβ/BMP growth factors are highly needed in applications in regenerative medicine and would allow to investigators address defined functionalities with minimal or no unwanted side effects.
